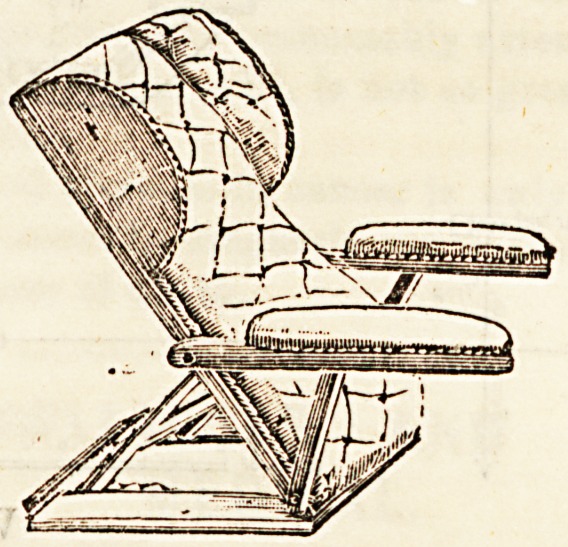# Bed Rest

**Published:** 1893-05-20

**Authors:** 


### BED REST.

We give below, also by kind permission of the same firm, a drawing of a very comfortable bed or back rest. The frame is made in polished mahogany or walnut, it is softly stuffed with hair and covered with cretonne. The arms are adjustable, and may be turned back if preferred or removed altogether. In cases of heart disease, asthma, &c., where the patient requires to be propped up in bed, a firm support of this kind is invaluable. More simple ones are made with merely a webbing back. Messrs. Farmer, Lane, and Co. have brought the production of these and other invalid appliances to a high state of perfection, and every article is thoroughly well made and carefully finished.

## Figures and Tables

**Figure f1:**